# Phylogenetic and Evolutionary Analysis Reveals the Recent Dominance of Ciprofloxacin-Resistant Shigella sonnei and Local Persistence of S. flexneri Clones in India

**DOI:** 10.1128/mSphere.00569-20

**Published:** 2020-10-07

**Authors:** Dhiviya Prabaa Muthuirulandi Sethuvel, Ankur Mutreja, Agila Kumari Pragasam, Karthick Vasudevan, Dhivya Murugan, Shalini Anandan, Joy Sarojini Michael, Kamini Walia, Balaji Veeraraghavan

**Affiliations:** a Department of Clinical Microbiology, Christian Medical College, Vellore, India; b Department of Medicine, University of Cambridge, Cambridge, United Kingdom; c Wellcome Sanger Institute, Hinxton, United Kingdom; d Division of Epidemiology and Communicable Diseases, Indian Council of Medical Research, New Delhi, India; University of Michigan—Ann Arbor

**Keywords:** antimicrobial resistance, multidrug resistance, Central Asia III, *Shigella*, pangenome

## Abstract

*Shigella* is the second leading cause of bacterial diarrhea worldwide. Recently, Shigella sonnei seems to be replacing Shigella flexneri in low- and middle-income countries undergoing economic development. Despite this, studies focusing on these species at the genomic level remain largely unexplored. Here, we compared the genome sequences of S. flexneri and S. sonnei isolates from India with the publicly available genomes of global strains. Our analysis provides evidence for the long-term persistence of all phylogenetic groups (PGs) of S. flexneri and the recent dominance of the ciprofloxacin-resistant S. sonnei lineage in India. Within S. flexneri PGs, the majority of the study isolates belonged to PG3 within the predominance of serotype 2. For S. sonnei, the current pandemic involves globally distributed multidrug-resistant (MDR) clones that belong to Central Asia lineage III. The presence of such epidemiologically dominant lineages in association with stable antimicrobial resistance (AMR) determinants results in successful survival in the community.

**IMPORTANCE** *Shigella* is the second leading cause of bacterial diarrhea worldwide. This has been categorized as a priority pathogen among enteric bacteria by the Global Antimicrobial Resistance Surveillance System (GLASS) of the World Health Organization (WHO). Recently, S. sonnei seems to be replacing S. flexneri in low- and middle-income countries undergoing economic development. Antimicrobial resistance in S. flexneri and S. sonnei is a growing international concern, specifically with the international dominance of the multidrug-resistant (MDR) lineage. Genomic studies focusing on S. flexneri and S. sonnei in India remain largely unexplored. This study provides information on the introduction and expansion of drug-resistant *Shigella* strains in India for the first time by comparing the genome sequences of S. flexneri and S. sonnei isolates from India with the publicly available genomes of global strains. The study discusses the key differences between the two dominant species of *Shigella* at the genomic level to understand the evolutionary trends and genome dynamics of emerging and existing resistance clones. The present work demonstrates evidence for the long-term persistence of all PGs of S. flexneri and the recent dominance of a ciprofloxacin-resistant S. sonnei lineage in India.

## Abstract (precis)

*Shigella* is the second leading cause of bacterial diarrhea worldwide. This has been categorized as a priority pathogen among enteric bacteria by the Global Antimicrobial Resistance Surveillance System (GLASS) of the World Health Organization (WHO). Recently, S. sonnei seems to be replacing S. flexneri in low- and middle-income countries undergoing economic development. Antimicrobial resistance in S. flexneri and S. sonnei is a growing international concern, specifically with the international dominance of the multidrug-resistant (MDR) lineage. Genomic studies focusing on S. flexneri and S. sonnei in India remain largely unexplored. This study provides information on the introduction and expansion of drug-resistant *Shigella* strains in India for the first time by comparing the genome sequences of S. flexneri and S. sonnei isolates from India with the publicly available genomes of global strains. The study discusses the key differences between the two dominant species of *Shigella* at the genomic level to understand the evolutionary trends and genome dynamics of emerging and existing resistance clones. The present work demonstrates evidence for the long-term persistence of all PGs of S. flexneri and the recent dominance of a ciprofloxacin-resistant S. sonnei lineage in India.

## INTRODUCTION

*Shigella* is ranked as the second leading cause of bacterial diarrhea worldwide and the third leading cause of death in children less than 5 years old (1). This has been categorized as a priority pathogen among enteric bacteria by the Global Antimicrobial Resistance Surveillance System (GLASS) of the World Health Organization (WHO) (2). Among the four species of *Shigella*, the most common cause of endemic shigellosis and the most frequently isolated species in India is Shigella flexneri, followed by S. sonnei. S. dysenteriae and S. boydii are now relatively uncommon. Studies from various parts of India have shown that the overall rate of isolation of *Shigella* species ranges from 3 to 6% among diarrheal patients (3). We have documented a prevalence of 4.8% to 4.6% between 2014 and 2015 and have continued to observe the same rate up to today at our tertiary care center in Vellore, India (4). In recent years, S. sonnei appears to be replacing S. flexneri in low- and middle-income countries undergoing economic development (5).

Several epidemics of *Shigella* have been reported from Asian countries such as Bangladesh, Sri Lanka, Maldives, Nepal, Bhutan, and Myanmar. In India, epidemics have been reported from Vellore, southern India; Andaman and Nicobar Islands, eastern India; and Chandigarh, northern India. Outbreaks caused by *Shigella* are not uncommon in India and have been reported from various parts of the country like West Bengal, Kerala, and Maharashtra. *Shigella* species are the third most common bacterial agents causing traveler’s diarrhea among enteric infections reported to FoodNet, and India is one of the most common travel destinations for picking up this infection (3).

Antimicrobial resistance (AMR) in S. flexneri and S. sonnei is a growing international concern, specifically with the international dominance of the multidrug-resistant (MDR) lineage of S. sonnei (6). A previous phylogenetic analysis predicted that South Asia would be the hub for the international spread of ciprofloxacin-resistant S. sonnei (6). However, only a limited number of *Shigella* isolates from India have been included in previous studies. Therefore, understanding the expansion of this lineage and the phylogenetic relationship with isolates from within and outside Asia, including India, is critical.

Genomic studies focusing on S. flexneri and S. sonnei in India remain largely unexplored. Understanding the genome sequences of antimicrobial-resistant pathogens can enhance our knowledge of the molecular identity of resistance traits and their mechanism of dissemination within the microbial population. Here, we compared the genome sequences of S. flexneri and S. sonnei isolates from India with publicly available genomes of global strains to understand the introduction and expansion of drug-resistant strains in India. Bayesian phylogenetic analysis was performed, in particular for S. sonnei isolates from India, to demonstrate the evolution of ciprofloxacin-resistant S. sonnei clones in India. In addition, virulence, resistance, and plasmid profiles of the isolates were analyzed and correlated with previously defined S. flexneri phylogenetic groups (PGs) and S. sonnei lineages.

## RESULTS

The whole-genome phylogenetic tree was constructed using 106 S. flexneri and 82 S. sonnei genomes sequenced in this study and 60 S. flexneri and 362 S. sonnei genomes from previous studies. All S. flexneri and S. sonnei sequences were mapped to the reference sequences (GenBank accession numbers CP000038 and NC_004337.2).

### Phylogenetic analysis of S. flexneri.

**FIG 1:**
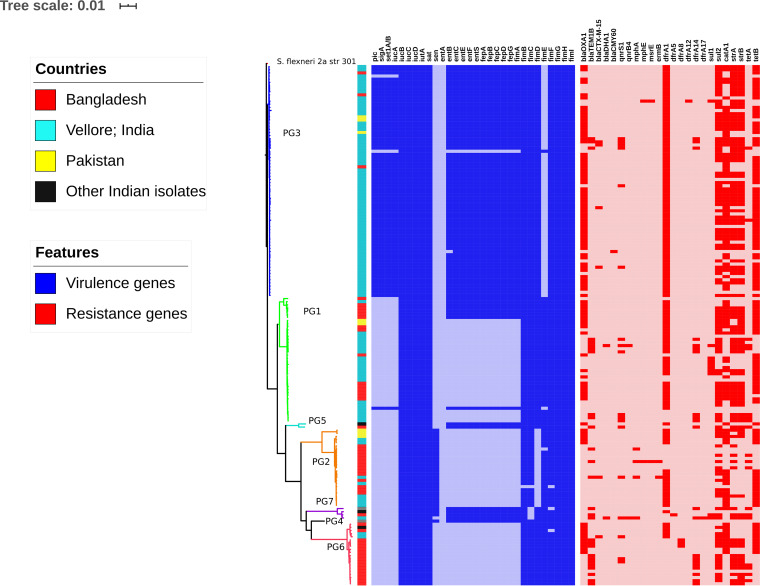
Maximum likelihood phylogenetic tree of 166 S. flexneri isolates mapped against the S. flexneri 2a strain 301 reference genome. The color strip indicates the location of isolates. The clade colors represent previously described phylogenetic groups (PGs). Virulence and antimicrobial resistance genes are represented as color gradients. The bar indicates the number of substitutions per site.

The phylogenetic comparison of Indian S. flexneri isolates against isolates available from other South Asian countries (Pakistan and Bangladesh) showed that Indian isolates were distributed across all the previously defined phylogenetic groups (PG1, -2, -3, -5, -6, and -7) with the exception of PG4 (Fig. 1). The majority of Indian isolates clustered within PG3 (70%), followed by PG1 (22%) and PG2 (7%). Each PG contained multiple serotypes. However, the common serotypes found within PG2, PG3, and PG5 were S. flexneri serotypes 3a, 2a, and 5a, respectively. The core single nucleotide polymorphism (SNP)-based comparisons between global and Indian strains suggested high genetic diversity within the S. flexneri PG3 isolates, with a median pairwise SNP difference of 97 SNPs (interquartile range [IQR], 88 to 105). The intragroup comparison of PG3 isolates from India showed a median difference of 78 SNPs (IQR, 47 to 100) (see Fig. S1 in the supplemental material).

10.1128/mSphere.00569-20.1 FIG S1 Average pairwise SNP distance per site comparison between intra- and intergroup isolates (red, intragroup; blue, intergroup). (a) Pairwise genetic distance between S. flexneri isolates. (b) SNP distances within S. flexneri PG3 between India and global isolates. (c) Pairwise genetic distance between S. sonnei isolates. (d) SNP distance within Central Asian lineage III of S. sonnei between India and global isolates. Download FIG S1, TIF file, 0.2 MB. Copyright © 2020 Muthuirulandi Sethuvel et al. 2020 Muthuirulandi Sethuvel et al. This content is distributed under the terms of the Creative Commons Attribution 4.0 International license.

Variation in the distribution of virulence determinants among the PGs was also evident. Notably, the SHI-1 pathogenicity island (PAI), which is known to carry *pic*, *sig*A, and the Shigella enterotoxin 1 (ShET-1) *set*1A and/or *set*1B genes, was exclusively seen in PG3 isolates with the exception of a single isolate of PG1. SHI-1 was present in various serotypes within PG3 but was predominantly seen in serotype 2a. Additionally, the SHI-2 PAI known to carry the *iuc*ABCD and *iut*A genes was found in isolates of all PGs. Similarly, *sat*, a serine protease autotransporter, was observed in all PGs. The *sen* gene encoding enterotoxin ShET-2 was seen only in the PG2 and PG7 isolates. Enterobactin genes (*ent*BCEFS and *fep*ABCDG) that encode iron siderophores were present across PG3, PG4, PG5, and PG7 and were also found in a few isolates of PG1. Notably, the *fim*E gene was absent in all PG3 isolates except one.

Screening of known AMR genes revealed highly variable AMR gene distributions both within and across PGs (Fig. 1). Genes encoding resistance to earlier first-line antimicrobials, including streptomycin (*str*A and/or *str*B), trimethoprim/sulfamethoxazole (*dfr*A1 and *sul*), chloramphenicol (*cat*A1), and tetracycline (*tet*A and/or *tet*B), were distributed across all the PGs. Among beta-lactamases, the *bla*_OXA-1_ gene was present in all PGs with the exception of PG4 and PG7. Similarly, *bla*_TEM-1B_ was present across all PGs with the exception of PG4, whereas *bla*_CTX-M-15_ was seen only in PG2, PG3, and PG6. The AmpC beta-lactamase gene *bla*_DHA_ was identified in two isolates, one each from PG1 and PG7. In addition, the *bla*_CMY-60_ gene was identified in PG3 alone. These AmpC genes were observed only in the Indian isolates. Furthermore, the analysis of fluroquinolone resistance mechanisms showed the presence of both plasmid-mediated quinolone resistance (PMQR) genes and mutations within the quinolone resistance-determining region (QRDR). The PMQR gene *qnr*S1 was identified in all PGs with the exception of PG4, while *qnr*B4 was seen in two isolates, one each from PG1 and PG7. The most frequently observed QRDR mutations were S83L in *gyr*A and S80I in *par*C as a result of replacements of serine by leucine and isoleucine, respectively. Macrolide resistance genes were recognized, such as *mph*A in PG2 and PG7, *erm*B and *mph*E in PG2, and *msr*E in PG3.

Additional plasmid analysis showed that IncFII was the most predominant plasmid across the PGs, followed by ColRNAI (Fig. 1). Among the Inc plasmid types, IncX3 was found in isolates belonging to PG1, PG2, and PG3, whereas IncY/IncL/M/IncI2 plasmids were identified only in PG3 isolates. Furthermore, IncFIC was seen only in PG1, while IncFIB was seen in all PGs with the exception of PG4.

### Phylogenetic analysis of S. sonnei.

**FIG 2:**
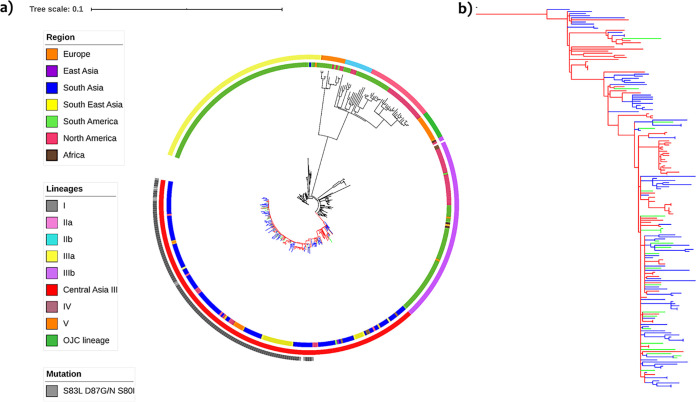
(a) Maximum likelihood phylogenetic tree of the Vellore ciprofloxacin-resistant S. sonnei isolates in a global context. The tree includes 446 S. sonnei sequences, including the reference sequence of S. sonnei Ss046. The clade color represents the Central Asia III lineage (blue, Vellore isolates; green, other Indian isolates; red, global isolates). The clades of the other lineages are in black. The innermost ring represents the region of the isolates, followed by the ring representing the lineages, and the outermost ring indicates QRDR mutations (triple mutant isolates). The bar indicates the number of substitutions per site. (b) Expanded view of the Central Asia III lineage.

The phylogenetic analysis of S. sonnei revealed that the ciprofloxacin-resistant isolates were grouped within the Central Asia III lineage. All Indian isolates, including those from Vellore, belonged to the Central Asia III lineage. The pairwise SNP comparison between global and Indian strains suggested high genomic identity within the S. sonnei Central Asia III lineage, with a median SNP difference of 8 SNPs (IQR, 5 to 11). The intragroup comparison of Central Asia lineage III isolates from India showed a median difference of 6 SNPs (IQR, 5 to 9) (Fig. S1). The majority of the isolates in the Central Asia III lineage carried genes encoding resistance to streptomycin, trimethoprim/sulfamethoxazole, and tetracycline, as described above. Most of the isolates (69%) within this lineage carried triple mutations (*gyr*A-S83L,D87G and/or D87N and *par*C-S80I) in the QRDRs (Fig. 2). Double and single mutations were observed in 1% and 19% of the isolates, respectively. Only one isolate carried a PMQR gene, *qnr*B. Central Asia lineage III had a different plasmid profile than the isolates of other lineages. Other lineages included ciprofloxacin-susceptible isolates with varying AMR and plasmid profiles. In addition, analysis of virulence determinants among the S. sonnei isolates showed a varying profile of virulence genes in comparison to S. flexneri. Notably, S. sonnei isolates were found to carry only the *sig*A gene within the SHI-1 PAI while harboring multiple virulence genes within the SHI-2 PAI. Furthermore, enterotoxin (*sen*) and enterobactin (*ent*BCEFS and *fep*ABCDG) genes were identified among the isolates.

**FIG 3:**
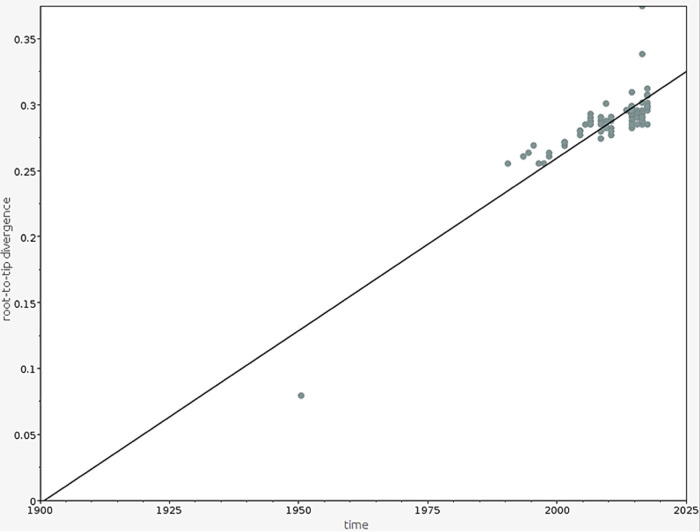
Root-to-tip branch lengths extracted from the maximum likelihood tree of the S. sonnei Central Asia III lineage from India plotted against the year of isolation.

**FIG 4:**
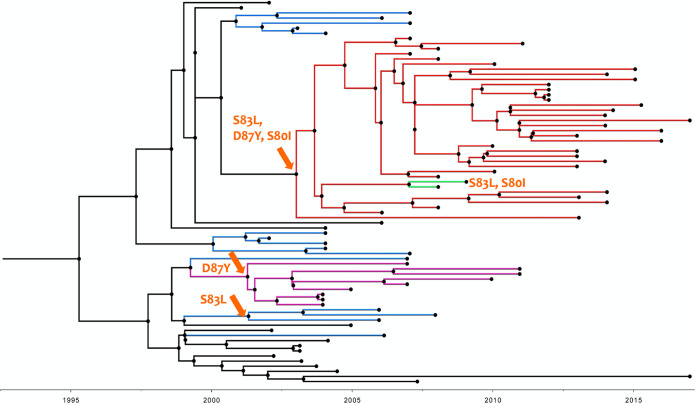
Temporal phylogenetic structure of 97 S. sonnei isolates belonging to the Central Asia III lineage from India (1990 to 2017). The orange arrows on the branches indicate the possible occurrence of specific QRDR mutations. Single, double, and triple mutations are in different colors.

As there was a strong temporal signature for the observed mutations in the global S. sonnei population, we explored the temporal structure of the S. sonnei isolates that belonged to the Central Asia III lineage from India using Bayesian phylogenetic methods. The root-to-tip analysis revealed a strong correlation (*R*^2^, 0.7543) between the time of isolation and distance from the root, suggesting temporal clocklike evolution in the S. sonnei Central Asia III lineage from India (Fig. 3). The median substitution rate of the S. sonnei population was estimated to be 2.083 × 10^−3^ substitutions per base per year in this study. This time-scaled phylogenetic reconstruction demonstrated the sequential accumulation of QRDR mutations among the S. sonnei population. The most recent common ancestor (MRCA) of the ciprofloxacin-resistant triple mutant clade in India was estimated to be from the year 2000 (95% highest posterior density [HPD], 1999.6 to 2004.4). Bayesian hierarchical clustering using core SNPs segregated the ciprofloxacin-resistant isolates that had a triple mutation in a QRDR into a separate clade (Fig. 4). Mutation *gyr*A-S83L was estimated to occur in ∼1996 (95% HPD, 1995 to 2001.1), mutation *gyr*A-D87Y was estimated to occur in ∼1996 (95% HPD, 1996 to 1999.8), and triple mutations S83L, D87Y/G, and S80I were estimated to occur in ∼2003 (95% HPD, 2003 to 2006.8). This implies that the ciprofloxacin-resistant population expanded rapidly after 2004 and has since been sustained.

Apart from fluroquinolone resistance, other antimicrobial resistance genes (ARGs) were also identified in the Central Asia III lineage. These included genes encoding resistance to previous first-line antimicrobials, such as *str*A/B, *tet*A/B, *dfr*A1, *sul*II, and *aph(3)-lb*/*aph(6)-Id*, conferring resistance to streptomycin, tetracyclines, trimethoprim, sulfonamide, and aminoglycosides, respectively. Other important ARGs were those belonging to extended-spectrum beta-lactamases (ESBLs) such as the *bla*_CTX-M_ and *bla*_TEM_ families. Three *bla*_CTX-M_ and two *bla*_TEM_ variants were identified; these included *bla*_CTX-M-15_, *bla*_CTX-M-14_, and *bla*_CTX-M-55_ as well as *bla*_TEM-1A_ and *bla*_TEM-1B_, respectively. Also, *mdf*A, an MDR transporter gene, was identified. In addition, we found the *mph*A gene, a macrolide resistance gene, among the Central Asia III S. sonnei isolates. Analysis of major plasmids among the isolates showed that Col-type, IncB/O/K/Z, IncFIA, and IncFIB plasmids were prevalent. Among the Indian Central Asia III S. sonnei isolates, genes conferring resistance to first-line antimicrobials and a single MDR transporter gene were widely seen, and the Col-type plasmid was the most common. The contrasting genomic features and epidemiological distributions of S. flexneri and S. sonnei are given in Table 1.

**TABLE 1:** Genomic and epidemiological features of S. flexneri versus S. sonnei

Feature	Description^*a*^	
	S. flexneri	S. sonnei
Geographical distribution(s)	Low- and middle-income countries	Industrialized countries

Multidrug resistance phenotype	AMP + SXT + NAL/CIP	AMP + SXT + NAL/CIP

Genotypic resistance determinant(s)		
Resistance gene(s)		
Trimethoprim/sulfamethoxazole	*dhfr*A1, *sul*I/*sul*II	*dhfr*A1, *sul*I/*sul*II
Ampicillin	*bla*_OXA-1_, *bla*_TEM-1_	*bla*_TEM-1_
Ciprofloxacin	QRDR mutations (*gyr*A and *par*C)	QRDR mutations (*gyr*A and *par*C)
	PMQR—*qnr*B/S and *aac(6′)-Ib-cr*	Efflux—*mdf*A, *acr*A/B, and *tol*C
	Efflux—*mdf*A, *acr*A/B, and *tol*C	
Cefotaxime/cefixime	*bla*_CTX-M-15/14_, *bla*_CMY2_, and *bla*_DHA_	*bla*_CTX-M-15/14_, *bla*_CMY2_, and *bla*_DHA_
Azithromycin	*mph*A/*erm*B	*mph*A/*erm*B
Mobile genetic elements		
Plasmid type	IncF	Col
Integron class(es)	1 and 2	2
PAI(s)	SHI-1, SHI-2, and SRL	SHI-2
Epidemiological feature(s)		
Existing terms	PGs	Lineages
Nature of spread	Limited global spread	Greater global spread
Dominant clone in India	PG3	Central Asia III lineage

a AMP, ampicillin; SXT, trimethoprim/sulfamethoxazole; NAL, nalidixic acid; CIP, ciprofloxacin; PAI, pathogenicity island; PGs, phylogenetic groups.

### Pangenome analysis.

The pangenome analysis of 106 S. flexneri strains identified 17,506 orthologs with 2,698 core genes (15%) and 14,808 accessory genes (85%). These included 452 soft core genes, 1,628 shell genes, and 12,728 cloud genes. A total of 9,845 unique genes were identified among the S. flexneri pangenome, with the FC533 strain belonging to PG3 having the largest number of unique genes (7,090). Of these genes, most of them were found in the accessory genome, and 26% of them were for hypothetical proteins.

**FIG 5:**
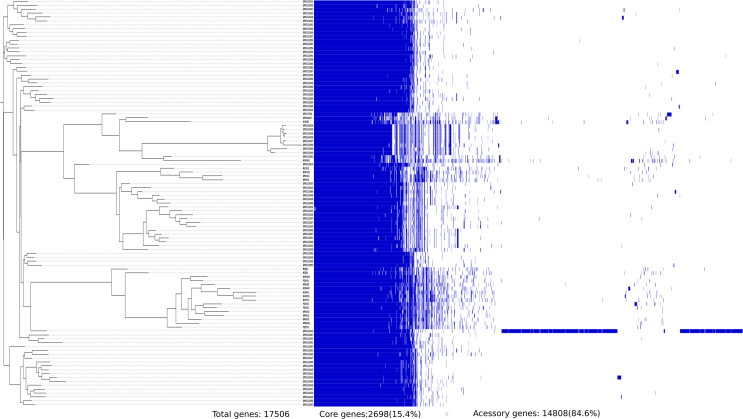
Core and accessory gene composition of 106 S. flexneri isolates extracted using Roary. A total of 2,698 core genes were shared by all strains, while 9,845 unique genes were identified.

**FIG 6:**
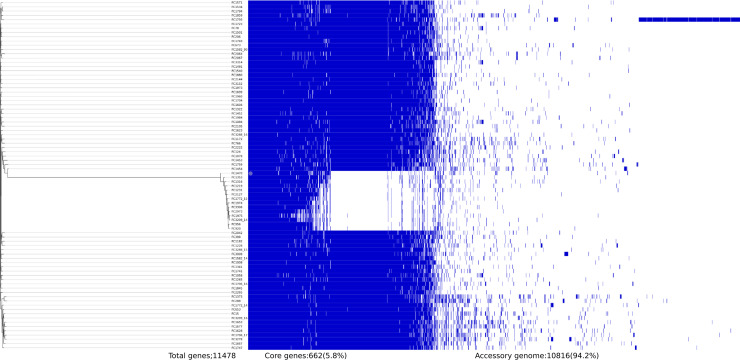
Core and accessory gene composition of 82 S. sonnei isolates extracted using Roary. A total of 662 core genes were shared by all strains, while 4,351 unique genes were identified.

In S. sonnei, the pangenome analysis of 82 isolates identified 11,478 orthologous groups with 662 core genes (6%) and 10,816 accessory genes (94%). These included 689 soft core genes, 3,262 shell genes, and 6,865 cloud genes. Notably, 14 isolates lost a few genes in their core genome and formed a separate cluster in the tree. The majority of these missing genes are found to encode hypothetical proteins (54%) with unknown functions and also include various transcriptional regulatory proteins. A total of 4,351 unique genes were identified, with the highest numbers (2,432) identified in the FC1793 strain belonging to the Central Asia III lineage. Like S. flexneri, most of the genes were found in the accessory genome, and 70% of them were for hypothetical proteins. The core and accessory gene compositions of S. flexneri and S. sonnei genomes are shown in Fig. 5 and 6.

## DISCUSSION

Here, we provide evidence for the long-term persistence of all PGs of S. flexneri and the recent dominance of the ciprofloxacin-resistant S. sonnei lineage in India. Unlike S. sonnei, the replacement of one particular S. flexneri PG over the other was not evident; instead, we saw that the older PGs have persisted along with the newer ones and continued causing disease, as reported previously. This was evidenced by the fact that every PG comprises at least one isolate collected since the early 1990s. Likewise, previous studies evidenced the longer-term colonization of S. flexneri in locations of endemicity with diverse populations of both antimicrobial-resistant and -susceptible strains cocirculating where selection for antimicrobial resistance is less favorable, thus resulting in substantially less international transmission (7, 8). The variation in the prevailing species in different settings might be due to specific transmission patterns (9).

The epidemiological species shift has been documented worldwide, mainly in Asian, Latin American, and Middle Eastern countries (5, 10). We reported an increasing S. sonnei prevalence in Vellore, from 20% in 2014 to 34% in 2015, in our previous study (4). Similar studies from other parts of India have also highlighted the rising dominance of S. sonnei in regions that were traditionally dominated by S. flexneri (11). Although the reasons behind this changing species-level epidemiology are poorly understood, a previous study by Thompson et al. presented that this could be due to several causes: (i) improved water quality prevents the passive immunization derived from Plesiomonas shigelloides, normally found in contaminated water; (ii) the amoeba Acanthamoeba castellanii has been shown to phagocytize S. sonnei efficiently and symbiotically and can thus withstand highly chlorinated environments where S. flexneri is not able to grow; and (iii) S. sonnei has a better ability to acquire antimicrobial resistance than S. flexneri (5).

In the present study, we have compared S. flexneri isolates mainly from three Southeast Asian countries for exploring the relationship of strains in this confined region. Regionally, S. flexneri appears to be highly endemic, and there is limited evidence of international dissemination reported within the last 30 years. Our phylogenetic analysis reveals that S. flexneri lineages are distinct and highly diverse compared to S. sonnei, as previously observed (9).

Previously, seven phylogenetic groups (PGs) of S. flexneri were defined by Connor et al. (9). Of the PGs, PG1, -2, -4, and -6 are reported to be the oldest lineages, while PG3 and PG5 are more recent. In this study, S. flexneri isolates were distributed across all the PGs except PG4 and were not specific to locations, unlike S. sonnei isolates, which are location specific and have wide global spread. The majority of the isolates belonged to PG3, which includes multiple serotypes of S. flexneri, with serotype 2 being predominant. This presence of multiple serotypes in a single PG shows that the core genome remains stable but that serotype switching has been common, as reported previously (9). The presence of different serotypes in a single cluster confirms phylogrouping as a more relevant method for public health surveillance and outbreak investigation than serotyping. This ensures that unless it is a protein-based vaccine carefully calibrated for stability across the phylogeny, vaccines against *Shigella* should continue to target the most common serotypes and not be lineage specific, as happens to be the case.

Recently, India developed the first indigenous vaccine against human shigellosis, licensed by the Indian Council of Medical Research (ICMR), India, and developed by the National Institute of Cholera and Enteric Diseases (NICED). OmpA of S. flexneri 2a coated with chitosan-alginate microparticles, which act as a delivery system, has been used as the vaccine target. OmpA of S. flexneri 2a was reported to be cross-reactive and induces strong protective immunity against prevalent serogroups of shigellae in animal models. Since the most prevalent species in India is found to be S. flexneri serotype 2, as observed in the present study and other studies in the country, this new vaccine could be a promising vaccine candidate against shigellosis in India if approved (12, 13).

Our study reveals variability in the composition of virulence factors among S. flexneri isolates. Notably, the copresence of the SHI-1 PAI, which was known to carry three essential virulence genes, along with enterobactin genes and antimicrobial resistance genes was exclusively observed in PG3, which is predominantly composed of S. flexneri serotype 2a isolates. This may account for the enhanced virulence and international dominance of this serotype. Furthermore, AMR has been shown to be a strong influence on the recent evolutionary history of many bacterial pathogens (7). The sustained presence of resistance genes for first-line antimicrobials in S. flexneri over the decades might be an essential factor for the successful maintenance of lineages in locations of endemicity, including India.

With regard to S. sonnei, there are five distinct lineages reported globally. The current pandemic involves globally distributed MDR clones that belong to lineage III. In particular, strains of the ciprofloxacin-resistant Central Asia III lineage are widespread. Although the Central Asia III lineage has spread internationally, its expansion in India has not been previously studied. Here, we compared Indian isolates against a global collection to provide insights into its expansion in our country. Our study provides evidence for the dominance of this lineage mainly in South India, as the other isolates from India included in the analysis have no location data available in the database. In addition, a few isolates from Africa, Europe, and America were found within this lineage and clustered closely with the Indian isolates, which indicates travel-related spread. Previous studies have also confirmed that India was the most commonly reported travel destination associated with ciprofloxacin-resistant S. sonnei and suggested that South Asia remains the primary source or hub for ciprofloxacin-resistant strains (6).

Furthermore, double/triple mutations in the QRDR in Central Asia lineage III isolates were consistent with previous findings and could be the reason behind the increased fitness and global expansion of this lineage alongside the overall synergy between PMQR *qnr* genes and chromosomal mutations that were noted in other studies from India (11). Interestingly, the copresence of *qnr* genes and mutations in *gyr*A and *par*C genes were identified in only one isolate in our study. Our data show the temporal introduction of the *gyr*A-S83L mutation in 1996 and the sequential accumulation of secondary mutations leading to the rapid expansion of ciprofloxacin-resistant clones within the Central Asia III lineage in the early 2000s. Our study also indicates that the earliest isolate (1990) within the Central Asia clade was from South Asia, which supports a previous hypothesis suggesting that South Asia was the likely origin of this lineage (6).

The emergence of resistance to previous first-line antimicrobials such as ampicillin, trimethoprim/sulfamethoxazole, and nalidixic acid during this intervening period made ciprofloxacin the drug of choice to treat drug-resistant shigellosis. Following this, due to the intensive use of ciprofloxacin, resistance to this drug increased from 0.6% in 1998 to 2000 to 29% in 2007 to 2009 in Asia and Africa (14). This further limited the treatment options and made third-generation cephalosporins and macrolides the drugs of choice. With the ESBL-producing strains that have left azithromycin as a last resort (1, 15, 16), the recent emergence of azithromycin resistance in S. sonnei and S. flexneri serotype 3a, particularly in men who have sex with men (MSM) communities, is no surprise (8).

Furthermore, the analysis of core and accessory genes of S. flexneri and S. sonnei revealed variation in composition. S. sonnei was found to have a larger accessory genome than S. flexneri. Strains FC533 and FC1793 were found to have the largest numbers of unique genes of the S. flexneri and S. sonnei strains compared, respectively. The core genome of S. flexneri remains stable, suggesting the strength of these genomes in adapting to evolutionary pressures for persistence, while few isolates of S. sonnei were missing certain core genes. Previous studies have shown that the loss of genes is a characteristic of an intracellular pathogenic lifestyle. In *Shigella*, genes that are more prone to deletion are generally associated with pathogenesis, and most of these deleted genes are found to be involved in cellular metabolism (17). Insights into these genes are out of the scope of this study and can be studied in the future; however, it would be interesting to assess whether this gene loss in S. sonnei provides any survival benefit to this pathogen.

While our study was limited by the number of *Shigella* sequences representing different regions of India, data from previous studies and this study show that *Shigella* species from different geographical locations share common AMR and virulence patterns. This indicates that the genetic contents of the isolates are based merely on the lineages circulating in the region. The presence of epidemiologically dominant lineages associated with stable AMR determinants results in successful survival in the community. The global dissemination of this lineage is more likely facilitated by frequent travel between other parts of the world.

## MATERIALS AND METHODS

### Bacterial isolates.

Totals of 106 S. flexneri and 82 S. sonnei strains isolated from stool specimens from patients with diarrhea or dysentery between 1990 and 2017 at the Department of Clinical Microbiology, Christian Medical College, Vellore, India, were included in the study. All isolates were retrieved from the archives and confirmed by a standard protocol (18). Serotype identification was done by the slide agglutination test using polyvalent and monovalent antisera (Denka, Seiken, Japan).

### Antimicrobial susceptibility testing.

Antimicrobial susceptibility testing of the isolates against ampicillin (10 μg), trimethoprim/sulfamethoxazole (1.25/23.75 μg), nalidixic acid (30 μg), norfloxacin (10 μg), cefotaxime (30 μg), cefixime (5 μg), and azithromycin (15 μg) was performed using the Kirby-Bauer disc diffusion method. The results were interpreted using breakpoints recommended by Clinical and Laboratory Standards Institute guidelines (19). The quality control strains used were Escherichia coli ATCC 35218 and E. coli ATCC 25922.

### Whole-genome sequencing.

Genomic DNA was extracted from a culture grown overnight using the QIAamp DNA minikit (Qiagen, Hilden, Germany) according to the manufacturer’s instructions. The quantity and quality of the DNA were analyzed using the Qubit 3.0 fluorometer (Thermo Fisher, USA) and the Nanodrop spectrophotometer (Thermo Fisher, USA). Paired-end genomic libraries were prepared with unique indexing of each DNA sample and sequenced using the short-read Illumina HiSeq V4 platform according to the manufacturer’s guidelines.

### Publicly available data.

S. flexneri and S. sonnei isolates obtained from human stool samples used in other whole-genome sequence analyses were included for comparative analysis in this study. The associated studies (along with the European Nucleotide Archive [ENA] study accession numbers) are as follows: discovery of sequence diversity in *Shigella* spp. (PRJEB2128), phylogeography of *Shigella* spp. (PRJEB5281), the *Shigella* CJ DB project (PRJEB2976), S. flexneri from around the world (PRJEB2542), global fluroquinolone-resistant S. sonnei genomic study (PRJNA320210), and intercontinental dissemination of azithromycin-resistant *Shigella* (PRJEB2846). Our study included representatives of S. flexneri phylogenetic groups (PGs) and S. sonnei lineages for analysis (see Table S1 in the supplemental material).

10.1128/mSphere.00569-20.2 TABLE S1 Metadata for S. flexneri and S. sonnei genomes used in this study for phylogenetic analysis. Download Table S1, XLSX file, 0.04 MB. Copyright © 2020 Muthuirulandi Sethuvel et al. 2020 Muthuirulandi Sethuvel et al. This content is distributed under the terms of the Creative Commons Attribution 4.0 International license.

### SNP-based phylogenetic analysis.

Phylogenetic analyses of S. flexneri and S. sonnei genomes were performed against publicly available global genomes. All S. flexneri and S. sonnei sequences were mapped to the S. flexneri 2a strain 301 and S. sonnei Ss046 reference genomes, respectively (GenBank accession numbers CP000038 and NC_004337.2), using SMALT (version 0.7.4), and SNPs were called against the reference and filtered using SAMtools (20). This resulted in alignments of 5,066 SNPs for S. sonnei and 67,454 SNPs for S. flexneri, which were used for phylogenetic inference. A maximum likelihood (ML) tree was constructed using RAxML v0.7.4 under the generalized time reversible (GTR) substitution model, and bootstrap replicates were determined (21). Pairwise SNP analysis was performed using pairwise_snp_differences (30).

The temporal signal in the ML phylogeny for S. sonnei was investigated using TempEst (http://tree.bio.ed.ac.uk/). The relationship between the root-to-tip distance and the time of isolation was analyzed. The temporal phylogenetic structure of S. sonnei was determined using Bayesian Evolutionary Analysis by Sampling Trees (BEAST) v.1.10 (22). The recombination-free core-genome SNP alignment file generated by Gubbins was used as the input to compute the mean evolutionary rate of the genomes and time of the most recent common ancestor (MRCA) (23). Trees were visualized in association with metadata using the Web-based Interactive Tree of Life (iTOL) (24). The study isolates were assigned to previously described lineages or PGs based on the clustering described previously by Connor et al. (9). The sequences were also analyzed for virulence, antimicrobial resistance (AMR), and mobile elements.

### Pangenome analysis.

Pangenome analysis of S. flexneri and S. sonnei isolates from India was performed using Roary v.3.11.2 with default settings (25). The genomes were annotated using Prokka (26). The genes that were common in all compared strains (core genes) and accessory genes were extracted and used to construct a phylogenetic tree. Core genes (99% ≤ strains ≤ 100%), soft core genes (95% ≤ strains < 99%), shell genes (15% ≤ strains < 95%), cloud genes (0% ≤ strains < 15%), and total genes (0% ≤ strains ≤ 100%) were calculated.

### Identification of virulence, resistance, and plasmids.

Genome data were analyzed for the presence of virulence determinants using VirulenceFinder 1.5 (27). Acquired antimicrobial resistance genes and chromosomal mutations in the QRDR were identified using ResFinder 2.1 (28) with a 90% threshold for identity and 60% minimum length coverage. The presence of plasmids was analyzed using PlasmidFinder 1.3 (29) with a 95% threshold for identity.

### Data availability.

We declare that all data that support the findings of this study are available within the paper (and its supplemental material) and from publicly available repositories. Raw sequences of Illumina reads generated from this study have been deposited in the ENA under project accession number PRJEB23045. Accession numbers for the sequences submitted to GenBank are provided in Table S1. All published tools used in this work are referenced in Materials and Methods. Further details are available from the corresponding author upon reasonable request.
