# Supplementary figures and images for: Phylogenetic and Evolutionary Analysis Reveals the Recent Dominance of Ciprofloxacin-Resistant Shigella sonnei and Local Persistence of S. flexneri Clones in India
Source: mSphere. 2020 Oct 7;5(5):e00569-20. doi: 10.1128/mSphere.00569-20 (PMC7568650; doi:10.1128/mSphere.00569-20)

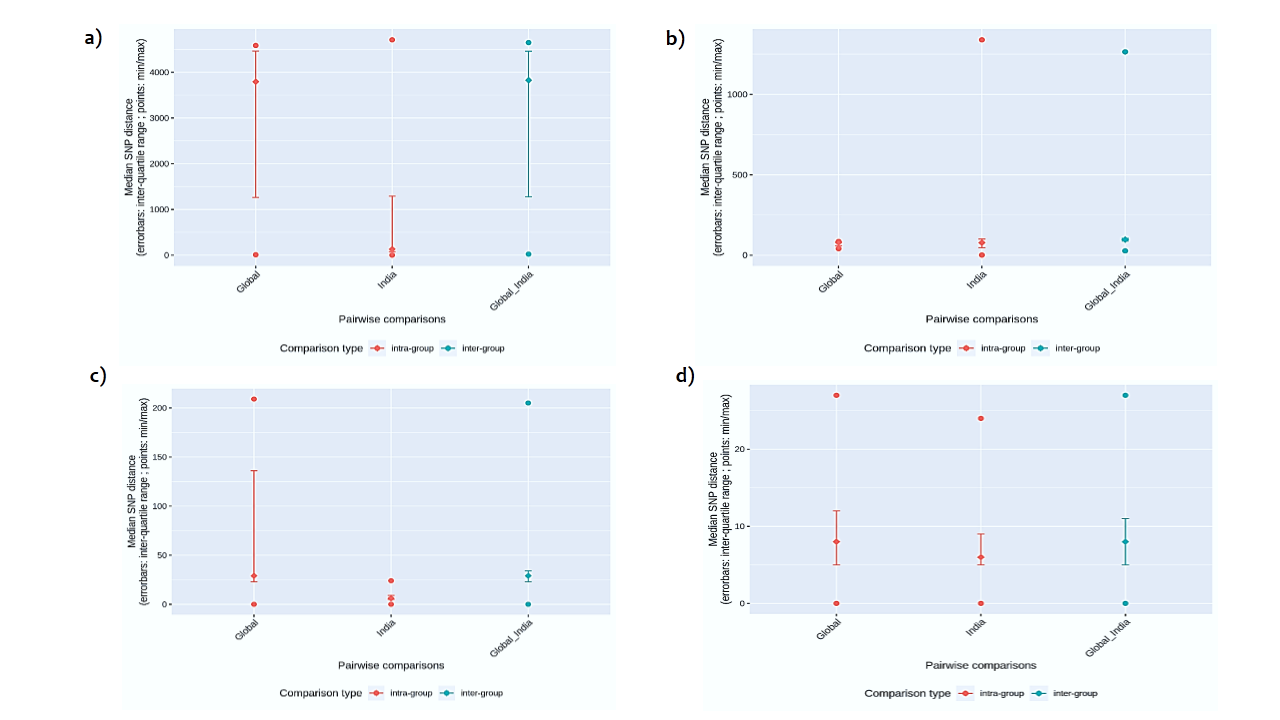

Supplement: FIG S1 [file mSphere.00569-20-sf001.tif]
